# Dietary *Coleus amboinicus* Lour. decreases ruminal methanogenesis and biohydrogenation, and improves meat quality and fatty acid composition in *longissimus thoracis* muscle of lambs

**DOI:** 10.1186/s40104-021-00654-3

**Published:** 2022-01-14

**Authors:** Yulianri Rizki Yanza, Malgorzata Szumacher-Strabel, Dorota Lechniak, Sylwester Ślusarczyk, Pawel Kolodziejski, Amlan Kumar Patra, Zora Váradyová, Dariusz Lisiak, Mina Vazirigohar, Adam Cieslak

**Affiliations:** 1 Department of Animal Nutrition, Faculty of Veterinary Medicine and Animal Science, Poznań University of Life Sciences, 60-637 Poznań, Poland; 2 Department of Biology Education, Universitas Islam Riau, Jl. Kaharuddin Nasution 113, Pekanbaru, 28284 Indonesia; 3 Department of Genetics and Animal Breeding, Poznań University of Life Sciences, Wołyńska 33, 60-637 Poznań, Poland; 4 Department of Pharmaceutical Biology and Botany, Wrocław Medical University, 50-556 Wrocław, Poland; 5 Department of Animal Physiology and Biochemistry, Faculty of Veterinary Medicine and Animal Science, Poznań University of Life Sciences, 60-637 Poznań, Poland; 6 Department of Animal Nutrition, West Bengal University of Animal and Fishery Sciences, Belgachia, K.B. Sarani 37, Kolkata, 700037 India; 7 Institute of Animal Physiology, Centre of Biosciences of Slovak Academy of Sciences, Šoltésovej 4-6, 040 01 Košice, Slovak Republic; 8 Department of Meat and Fat Technology, Wacław Dąbrowski Institute of Agricultural and Food Biotechnology, Rakowiecka 36, 02-532 Warszawa, Poland; 9 Zist Dam Group, University Incubator Center, University of Zanjan, Zanjan, 45371-38791 Iran

**Keywords:** Bioactive compounds, Biohydrogenation, Meat characteristics, Methane, Microorganism, Ruminal fermentation, Sheep

## Abstract

**Background:**

Methane production and fatty acids (FA) biohydrogenation in the rumen are two main constraints in ruminant production causing environmental burden and reducing food product quality. Rumen functions can be modulated by the biologically active compounds (BACs) of plant origins as shown in several studies e.g. reduction in methane emission, modulation of FA composition with positive impact on the ruminant products. *Coleus amboinicus* Lour. (CAL) contains high concentration of polyphenols that may potentially reduce methane production and modulate ruminal biohydrogenation of unsaturated FA.

This study aimed to investigate the effect of BAC of *Coleus amboinicus* Lour. (CAL) fed to growing lambs on ruminal methane production, biohydrogenation of unsaturated FA and meat characteristics. In this study, the in vitro experiment aiming at determining the most effective CAL dose for in vivo experiments was followed by two in vivo experiments in rumen-cannulated rams and growing lambs. Experiment 1 (RUSITEC) comprised of control and three experimental diets differing in CAL content (10%, 15%, and 20% of the total diet). The two in vivo experiments were conducted on six growing, rumen-cannulated lambs (Exp. 2) and 16 growing lambs (Exp. 3). Animals were assigned into the control (CON) and experimental (20% of CAL) groups. Several parameters were examined in vitro (pH, ammonia and VFA concentrations, protozoa, methanogens and select bacteria populations) and in vivo (methane production, digestibility, ruminal microorganism populations, meat quality, fatty acids profiles in rumen fluid and meat, transcript expression of 5 genes in meat).

**Results:**

CAL lowered in vitro methane production by 51%. In the in vivo Exp. 3, CAL decreased methane production by 20% compared with the CON group, which corresponded to reduction of total methanogen counts by up to 28% in all experiments, notably Methanobacteriales. In Exp. 3, CAL increased or tended to increase populations of some rumen bacteria (*Ruminococcus albus, Megasphaera elsdenii, Butyrivibrio proteoclasticus*, and *Butyrivibrio fibrisolvens*). Dietary CAL suppressed the Holotricha population, but increased or tended to increase *Entodiniomorpha* population in vivo*.* An increase in the polyunsaturated fatty acid (PUFA) proportion in the rumen of lambs was noted in response to the CAL diet, which was mainly attributable to the increase in C18:3 *cis*-9 *cis*-12 *cis*-15 (LNA) proportion. CAL reduced the mRNA expression of four out of five genes investigated in meat (fatty acid synthase, stearoyl-CoA desaturase, lipoprotein lipase, and fatty acid desaturase 1).

**Conclusions:**

Summarizing, polyphenols of CAL origin (20% in diet) mitigated ruminal methane production by inhibiting the methanogen communities. CAL supplementation also improved ruminal environment by modulating ruminal bacteria involved in fermentation and biohydrogenation of FA. Besides, CAL elevated the LNA concentration, which improved meat quality through increased deposition of n-3 PUFA.

## Highlight

*• Coleus amboinicus* Lour. (CAL) into sheep diet decreased CH_4_ emission.

*•* CAL did not reduce nutrient digestibility, but inhibited the methanogen community.

*•* CAL increased ruminal propionate proportion and decreased acetate/propionate ratio.

*•* CAL elevated n-3 fatty acid concentration in ruminal fluid and meat.

*•* Supplementation of CAL improved some meat quality traits.

**Graphical abstract:**

## Graphical abstract

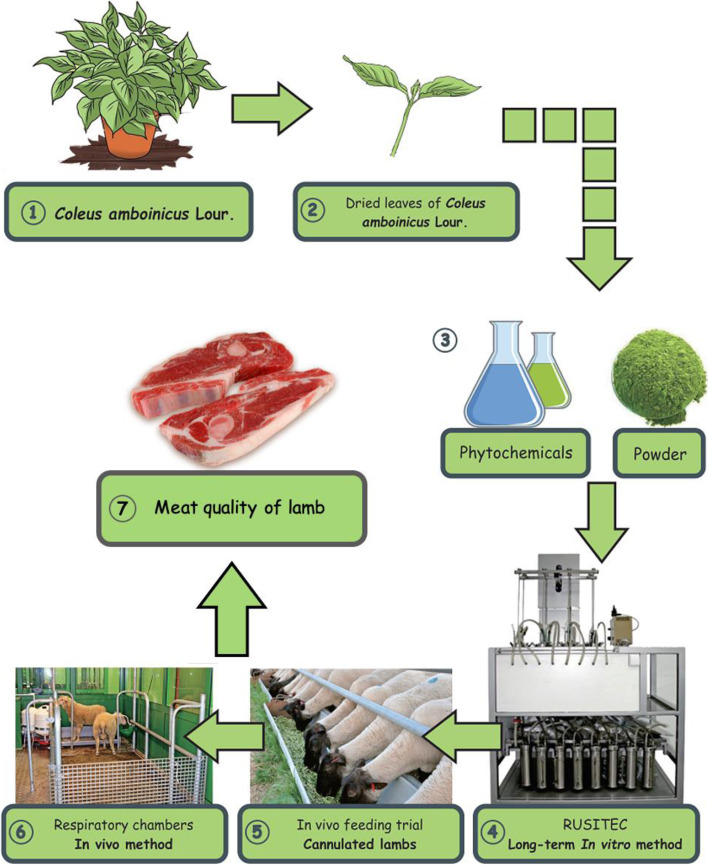

## Introduction

Methane (CH_4_) is a greenhouse gas mainly produced by anaerobic enteric fermentation in the rumen. The enteric CH_4_ production also represents a loss of the total energy (2–12%) decreases the efficiency of animal production [1]. Enteric CH_4_ emission is thus one of the main targets of greenhouse gas mitigation efforts to reduce CH_4_ production in the animal sector.

Biologically active compounds (BACs) have been recognized as modulators of rumen microbial fermentation, including methanogenesis [2, 3]. Thus, the BACs can reduce the negative animal impact on the environment such as enteric methane emission [4]. The use of BACs may also modulate the ruminal biohydrogenation (BH) of unsaturated fatty acids (UFAs), causing changes in the fatty acid (FA) profile of the ruminal fluid and consequently of ruminant products. Reduction of ruminal CH_4_ production should be balanced with improvements in rumen performance, and enrichment of animal products with beneficial FAs for sustainable adoption of CH_4_ mitigation technologies in the livestock industry [1, 5]. Researchers are still searching for the most effective sources and doses of BACs that could be recommended for long-term application.

One category of BACs are polyphenols, such as phenolic acids, flavonoids, condensed tannins and hydrolysable tannins [3]. *Coleus amboinicus* Lour. (CAL) is rich in polyphenolic compounds (mostly phenolic acids and flavonoids), diterpenes, and alkaloids [6, 7]. *C. amboinicus* grows in tropical regions, including Asia, Africa and Australia, and is used in human medicine for long time [7]. Our previous short-term in vitro study revealed the capacity of CAL to decrease CH_4_ production and to modulate ruminal FA composition, mainly n-3 polyunsaturated fatty acids (PUFAs) by altering the microbial activity linked to methanogenesis and FA biohydrogenation [6]. However there is no published evidence documenting effect of polyphenol-rich CAL on ruminal fermentation by a long-term in vitro study or by an in in vivo experiment.

Therefore, this study investigated the effects and mode of action of BACs present in CAL on rumen methanogenesis and BH in growing lambs through a long-term in vitro and two in vivo experiments. We hypothesized that CAL could 1) affect rumen microbial population (mainly methanogens) and consequently mitigate ruminal CH_4_ production, and 2) modulate the BH of UFA, especially n-3 PUFAs, in the rumen and animal tissues, presumably, without any negative effect on rumen parameters or animal performance.

## Materials and methods

### Experimental design

The CAL used in this study were purchased from a commercial source (Karya Herbal Nasional Ltd., company land-plot at Bogor, Indonesia 6°70′28″S; 106°90′90″E and 6°43′30.1″S; 107°05′09.2″E). The CAL were randomly collected after 2 to 3-month growth period and dried in an oven at 50–60 °C for 48 h. The leaves were ground and prepared for analyses.

### In vitro experiment

The Exp. 1 was carried out using the long-term in vitro rumen simulation technique (RUSITEC) following the procedures described by Kozłowska et al. [8]. The RUSITEC experiment was designed with a completely randomized block design with four diets and two replicates in each run, and repeated three times. The four isonitrogenous and isoenergetic diets were prepared as follows: diet 1: a control diet CON based on grass silage (5.7 g DM) and concentrate (5.3 g DM); diet 2: 10% CAL (6.4 g DM of grass silage, 3.5 g DM of concentrate, and 1.1 g DM of CAL); diet 3: 15% CAL (6.5 g DM of grass silage, 2.85 g DM of concentrate, and 1.65 g DM of CAL diet), and diet 4: 20% CAL (6.5 g DM of grass silage, 2.3 g DM of concentrate, and 2.2 g DM of CAL). The diets for animals donating the rumen fluids as well as animals for in vivo experiments were formulated using the IZ INRA [9] system to meet the animals’ nutrient requirements (average for 20 kg of body weight and 150 g/d of growth: 0.72 unit for meat production per day and 69 g protein truly digestible in the small intestine per day). The chemical composition of the grass silage, concentrate, and CAL are presented in Table 1. Rumen fluid and solid digesta for the in vitro experiment diet were collected before morning feeding from six rumen-cannulated lambs (20 ± 3 kg) for microbial inocula. The lambs, donors of the rumen fluid, were fed the same diet as in the CON treatment.

**Table 1:** Chemical composition and fatty acids profile of dietary components and CAL

Item	Grass Silage	Concentrate	CAL
Dry matter content, g/kg	416	889	919
Chemicals composition, g/kg DM			
Ash	86.9	71.9	153
Organic matter	913	928	847
Crude protein	187	203	214
Ether extract	20.6	38.0	43.3
aNDF	456	238	405
Fatty acids, g/100 g FA			
C14:0	0.90	0.20	0.45
C16:0	19.9	16.3	18.7
C18:0	5.88	3.71	4.35
C18:1 *cis-* 9	8.80	23.7	2.50
C18:2 *cis-* 9. *cis-* 12	14.4	42.7	10.8
C18:3 *cis-* 9 *cis-* 12 *cis-* 15	36.1	9.20	45.1
∑ Other FA	13.9	4.23	18.1
∑ SFA	30.1	21.4	26.0
∑ UFA	69.9	78.6	74.0
∑ MUFA	14.3	26.2	14.8
∑ PUFA	55.5	52.5	59.2
∑ n-6 FA	17.7	43.0	12.3
∑ n-3 FA	37.8	9.42	46.9

*CAL C. amboinicus* Lour.*, DM* dry matter, *aNDF* NDF analyzed with α-amylase, *FA* fatty acids, *SFA* saturated fatty acids, *UFA* unsaturated fatty acids, *MUFA* monounsaturated fatty acids, *PUFA* polyunsaturated fatty acids

Samples of fermentation fluid were collected directly from each fermenter 3 h before replacing the bags with the diets. The pH, ammonia concentration, VFA profile, feed degradability, protozoa count, and populations of methanogen, total bacteria, and select bacteria were analyzed. To determine FA profile, samples of the fermenting fluid were directly collected from the effluent vessels while the bags were being replaced. Fermentation gases were collected over 24 h using gas-tight bags (Tecobag 81, Tesseraux Container, Bürstadt, Germany).

### In vivo experiment

The Exp. 2 employed six rumen-cannulated lambs allocated into two treatments, i.e., the control diet (CON) and the experimental diet (CAL-containing diet) in a crossover design. The highest level of CAL (20%) was selected based on the in vitro results from RUSITEC experiment. Lambs on CON diet received 352 g DM/d of concentrate and 379 g DM/d of grass silage. The experimental lambs received concentrate, CAL and grass silage. During the first 14 d of the experiment, the concentrate (352 g/d) in the experimental diet was gradually replaced by CAL from 46 g DM/d to 173 g DM/d. From d 15, the experimental lambs received 176 g DM/d of concentrate, 173 g DM/d of CAL and 507 g DM/d of grass silage. The CAL dose of 173 g DM/d corresponded to 20% of CAL in daily diet intake (856 g DM/d). In Exp. 2, each period lasted 24 d, with a 21-day adaptation period and a 3-day sampling period. Rumen fluid from each lamb was collected daily for 3 d of the experimental period, before morning feeding (0 h), and then at 3 h and 6 h after morning feeding [8]. The pH, ammonia concentration, VFA profile, and numbers of protozoa, methanogens, and total bacteria were analyzed. Meanwhile, samples for quantification of total bacteria and methanogens using fluorescence in situ hybridization (FISH) were only collected at the 3 h timepoint.

In Exp. 3, sixteen growing lambs (20 ± 3 kg live weight) were used for the final production performance test. Lambs were randomly allocated into CON or CAL dietary treatments based on their live weight (*n* = 8 per group). Lambs were kept individually during the whole experiment, except during the period when respiratory chambers were used. In order to reduce stress associated with isolation, two animals were always kept together in each cage placed in the respiratory chamber. The experiment lasted 30 d, a 21-day adaptation stage and an 8-day sampling period, with 1-day for the slaughtering process. During the adaptation period, the lambs were adapted to the CAL diet, as in Exp. 2. The CON lambs were fed the control diet comprised of grass silage (379 g DM/d) and concentrate (352 g DM/d). The CAL lambs were fed grass silage (507 kg DM/d), concentrate (176 g DM/d), and CAL (173 g DM/d). The diets were formulated following the IZ INRA [9] system to meet animals’ major nutritional requirements. All animals had free access to fresh water. The CON and CAL diets were fed in equal proportion at 8:00 h and 20:00 h daily. Feed intake, feed residue, and amount of feces were recorded daily. Animal weights were recorded weekly. During the sampling period (from d 22 to d 28 of the experiment), each cage was transferred into a respiratory chamber by daily rotation in order to determine the direct CH_4_ emission for 24 h consecutively. Two respiratory chambers were used. Each cage was tested twice but in order to obtain individual lamb’s gasses production, obtained results were divided by two.

On the last day of experiment (d 30), the animals were slaughtered 3 h after morning feeding. After slaughtering, the rumen digesta were taken from the top, bottom, and middle of the rumen and squeezed through a four-layer cheese-cloth for analysis of pH, ammonia concentration, and VFA profile in ruminal fluid, FA profile, and populations of protozoa, methanogens, total bacteria, and select bacteria in digesta. Samples of muscle from the right side of each carcass and drawn at the level of the thirteenth thoracic rib was immediately collected. Approximately 5 g of *longissimus thoracis* (LT) muscle was shock-frozen in liquid nitrogen for gene expression analysis. The LT muscle (ca. 50 g) was cooled and transferred in a cool (4 °C) atmosphere to the laboratory for FA analysis. All collected samples were stored at − 80 °C until analysis.

### Meat quality traits

The LT (100 g) from the right-half carcass was used for meat quality analysis that was performed at the laboratory of the Institute of Agricultural and Food Biotechnology (Poland). The pH was measured in triplicates 24 h post-mortem on samples of LT muscle using a pH meter with an integrated electrode (pH meter 1140, Mettler-Toledo, USA) following ISO 2917 (2001) instructions. For the instrumental evaluation of meat color, 10 mm thick steaks of LT muscle were cut towards the direction of muscle fibers and exposed to electric light for 15 min. The values of L* (lightness), a* (redness), and b* (yellowness) were determined in triplicates using a Minolta Chroma Meter CR- 400 (Konica-Minolta, Japan). Compositional analysis of LT muscle (water, intramuscular fat (IMF) and total protein content) was performed using minced samples according to the methods described in ISO 1442 (2000) for water, ISO 1444 (2000) for fat (using a Soxtherm device, Gerhardt Analytical System, Germany), and PN-75/A-04018 (2000) for protein (using a Kjeltec System 1002 Distilling Unit, FOSS Analytical, Denmark). The water-holding capacity (WHC) of minced LT muscle samples was determined as described by Grau and Hamm [10], with later modifications introduced by Pohja and Ninivaara [11]. Visual evaluations of meat color and marbling of LT muscle samples were performed by a panel of four assessors using a 1–8 point Soicarni scale for meat color, with 1 being the lightest and 8 the darkest color, and a 1–4 point scale for marbling (developed by the Institute of Agricultural and Food Biotechnology, Poland), with 1 being related to minor and 4 to the greatest marbling. The taste panel of four professional assessors, trained in rating lamb for meat-eating quantity, was used to assess aroma, juiciness, tenderness, and flavor on boiled LT samples. Assessors scored the samples for each trait separately on a 1–5 point scale, where 1 was related to bad and 5 to a very good level of the traits according to the methodology of Barylko-Pikielna [12]. Concerning the above mentioned visual and sensory evaluations of meat, the mean values of the scores given by four assessors were taken for further calculations.

### Determination of phenolic acid, flavonoid, and diterpenoid contents

The CAL preparation and extraction were caried out as described previously [7]. All the analyses were performed in triplicate for three independent samples that were stored in a freezer at − 20 °C before analysis. The CAL bioactive compounds (phenolic acid, flavonoid, and diterpenoid) were analyzed by ultra-high-resolution mass spectrometry (UHRMS) on Dionex UltiMate 3000RS system (Thermo Scientific, Darmstadt, Germany) with a charged aerosol detector interfaced with a high-resolution quadrupole time-of-flight mass spectrometer (HR/QTOF/MS, Compact, Bruker Daltonik, Bremen, Germany) according to the procedure of Ślusarczyk et al. [7].

### Determination of the chemical composition of feeds

Samples of grass silage, concentrate, CAL, and feces were analyzed according to AOAC [13] for DM (method no. 934.01), ash (method no. 942.05), crude protein (CP; using a Kjel-Foss Automatic 16,210 analyzer; method no. 976.05), and ether extract (EE, using a Soxhlet System HT analyzer; method no. 973.18). The organic matter (OM) content was calculated by subtracting ash concentration from DM content. The aNDF was determined following the method of Van Soest et al. [14], with the addition of amylase and sodium sulfite without residual ash.

### Basic rumen fermentation analysis and CH_4_ measurement

The pH of ruminal samples from all experiments was measured immediately after samples collection using a pH meter (CP-104; Elmetron, Zabrze, Poland). The ammonia concentration was analyzed using the colorimetric Nessler method described earlier by Bryszak et al. [5]. The VFA profile was determined by gas chromatography (GC Varian CP 3380, Sugarland, TX, USA) following the protocol of Varadyova et al. [15]. The in vitro CH_4_ concentration was measured using a gas chromatography in SRI PeakSimple model 310 (Alltech, PA, USA) following the procedure described by Kozłowska et al. [8]. Methane production in the in vivo experiment was measured using two respiration chambers (SPA System, Wrocław, Poland). The total chamber volume (8.2 m^3^) was ventilated by recirculating fans set at 40 m^3^/h giving approximately 5 air changes per hour. The temperature and relative humidity were set at 16 °C and 60%, respectively. The concentrations of CH_4_ and CO_2_ were measured using two nondispersive infrared spectroscopy detectors operating in the near-infrared spectrum (Servomex 4100, Servomex, UK; 1210 Gfx detector). Measurements were taken at two-second intervals. Two measuring channels were used: the concentration of CO_2_ in the range of 0–2.5% (0–48, 450 mg/m^3^) and the CH_4_ concentration in the range of 0–1000 ppm (0–706 mg/m^3^). The sample was collected and then ducted to the analyzer via a polyethylene tube with a diameter of 8 mm. The sampling rate was 0.6 L/min. Before starting the experiment, the analyzers were calibrated using as calibration gases (99.999% nitrogen gas by volume, 1210 ppm CH_4_ in nitrogen, and 4680 ppm CO_2_ in nitrogen). The analyzer was equipped with a 0.17 L cuvette with an optical track of 540 mm for CH_4_ and a 0.012 L cuvette with an optical track length of 154 mm for CO_2_.

### Microbial quantification

The protozoa population was quantified following the method described by Michalowski et al. [16]. Methanogen numbers were quantified by fluorescence in situ hybridization (FISH) technique according to the procedure of Yanza et al. [6]. For bacteria quantification, total DNA was extracted from fermented fluid using QIAamp DNA Stool mini kit (Qiagen GmbH, Hilden, Germany) according to Yanza et al. [6]. Sequences of primers specific to the particular bacterial species or genera are presented in Table 2 [17–24]. Bacteria quantification was performed with a QuantStudio 12 Flex PCR system (Life Technologies, Grand Island, NY, USA).

**Table 2:** The sequences of primers specific to the analyzed bacteria species

Species	Primer sequences (5′ to 3′)	Reference
*Streptococcus bovis*	F: TTCCTAGAGATAGGAAGTTTCTTCGG	[17]
	R: ATGATGGCAACTAACAATAGGGGT	
*Ruminococcus flavefaciens*	F:CGAACGGAGATAATTTGAGTTTACTTAGG	[18]
	R: CGGTCTCTGTATGTTATGAGGTATTACC	
*Ruminococcus albus*	F: CCCTAAAAGCAGTCTTAGTTCG	[19]
	R: CCTCCTTGCGGTTAGAACA	
*Megasphaera elsdenii*	F: AGATGGGGACAACAGCTGGA	[17]
	R: CGAAAGCTCCGAAGAGCCT	
*Prevotella* spp.	F: GAAGGTCCCCCACATTG	[17]
	R:CAATCGGAGTTCTTCGTG	
*Lactobacillus* spp.	F: TATGGTAATTGTGTGNCAGCMGCCGCGGTAA	[20]
	R: AGTCAGTCAGCCGGACTACHVGGGTWTCTAAT	
*Fibrobacter succinogenes*	F: GTTCGGAATTACTGGGCGTAAA	[21]
	R: CGCCTGCCCCTGAACTATC	
*Butyrivibrio proteoclasticus*	F: TCCTAGTGTAGCGGTGAAATG	[22]
	R: TTAGCGACGGCACTGAATGCCTA	
*Butyrivibrio fibrisolvens*	F: ACACACCGCCCGTCACA	[23]
	R: TCCTTACGGTTGGGTCACAGA	
*Anaerovibrio lipolytica*	F: GAAATGGATTCTAGTGGCAAACG	[24]
	R:ACATCGGTCATGCGACCAA	

### Analysis of fatty acid in feed and meat samples

The FA profiles of the grass silage, concentrate, CAL, rumen fluid, and LT muscle were analyzed following the procedure of Bryszak et al. [5]. Sample hydrolysis was carried out in a closed system using screw-cap Teflon-stoppered tubes (Pyrex, 15 mL). Three milliliters of 2 mol/L NaOH was added to 100, 2500, 100, 10, and 500 mg of grass silage, concentrate, CAL, rumen fluid, and meat samples, respectively A gas chromatograph (GC Bruker 456-GC, USA) fitted with a flame ionization detector and a 100 m fused-silica capillary column (0.25 mm i.d.) coated with 0.25 μm Agilent HP (Chrompack CP7420) were used. The conjugated linoleic acid (CLA) peaks were identified via comparison with the retention times of the reference standard (conjugated linoleic acid methyl esters, and a mixture of cis- and trans-9, − 11 and − 10,12-octadecadienoic acid methyl esters; Sigma) using Galaxie Work Station 10.1 (Varian, CA, USA). The desaturase index, atherogenic index, and thrombogenic index were calculated as described by Bryszak et al. [5].

### Analysis of mRNA expression in meat samples

Transcript analysis of *FADS1, FASN, LPL, SCD,* and *ELOVL5* genes in the meat samples was performed using quantitative PCR (qPCR) analysis. Total RNA was isolated from 100 mg of LT muscle using Extrazol reagent. In brief, the meat samples were homogenized in 0.5 mL of Extrazol reagent using a TissueLyser II (Qiagen, USA). After 10 min incubation, 200 μL of chloroform was added and shaken vigorously for 15 s. The samples were then incubated for 10 min at room temperature and centrifuged for 15 min at 12,000 × *g*. Next, the upper aqueous phase was transferred to a new tube and 0.5 mL of isopropanol was added. The samples were again incubated and centrifuged as in the previous step. The resulting RNA pellet was washed with 1 mL of 75% EtOH and dissolved in RNAse free water (Sigma Aldrich). The quantity and quality of the isolated total RNA was checked using an NP80 NanoPhotometer (Implen, Germany). A reverse transcription reaction (RT) was carried out with 1 μg of total RNA and the Firescript RT cDNA Synthesis MIX with Oligo (dT) and Random primers (Solis BioDyne), following the manufacturer’s protocol. The mRNA expression was quantified using QuantStudio 12 Flex PCR system (Life Technologies, Thermo Fisher Scientific, Waltham, MA, USA) and SYBR Green PCR Master Mix (Thermo Fisher Scientific, Waltham, MA, USA). The primer pairs used for RT-qPCR amplification are listed in Table 3 [25]. The specificity of reaction products was determined by the melting points (0.1 C/s transition rate). Two genes have been considered as reference, *GAPDH* and β-actin. Due to its higher stability, the β-actin gene was applied and relative mRNA expression was evaluated by delta-delta CT (ΔΔCT).

**Table 3:** The sequences of primers specific to the analyzed genes expression in the *longissimus thoracis* muscle of lambs

Gene name	Primer sequence (5′ to 3′)		Reference
	Forward	Reverse	
*SCD*	GAGTACCGCTGGCACATCAA	CTAAGACGGCAGCCTTGGAT	[25]
*ELOVL5*	TGCTTCAGTTTGTGCTGACC	TGGTCCTTCTGGTGCTCTCT	[25]
*FASN*	GGAGGACGCTTTCCGTTACA	TGCTCTTCCTCACGTACCTGAA	[25]
*FADS1*	CTGCTGTACCTGCTGCACAT	ACGGACAGGTGTCCAAAGTC	[25]
*LPL*	TCATCGTGGTGGACTGGCT	CATCCGCCATCCAGTTCATA	[25]

Analyzed expression of five genes: *SCD* stearoyl-CoA desaturase, *ELOVL5* fatty acid elongase 5, *FADS1* fatty acid desaturase 1, *FASN* fatty acid synthase and *LPL* lipoprotein lipase

### Statistical analysis

The data of the experiment 1 (RUSITEC) were analyzed using a mixed model procedure (PROC MIXED) in SAS (university edition, version 9.4; SAS Institute, Cary, NC, USA) with repeated measures of day and fermenter treated as the experimental unit. The dietary treatment was considered as the fixed effect, experimental run as the random effect, and the day (6 to 10 d) as the repeated factor. Differences among treatments were further determined using Tukey’s post hoc test and linear orthogonal contrast was used to ascertain the tendency of the dose effect of CAL. In experiment 2, data were analyzed using PROC MIXED of SAS with the model containing dietary group, hour, and their interaction (group × h) as the fixed effects and the animal and hour of sample collection as the random effect with repeated measures. When the significant value of the interaction occurred, Tukey’s post hoc test was used to estimate the differences between means. In experiment 3, data were analyzed using PROC TTEST procedure of SAS, and for all parameters each animal was considered as the experimental unit. Significance was accepted at *P* < 0.05 and tended to significance at 0.05 < *P* < 0.10. All the values are shown as group means with pooled standard errors of means.

## Results

### Nutrients and phytochemical composition of CAL

The CAL had higher CP, EE, and ash concentrations than the concentrate and grass silage (Table 1). It also had relatively higher PUFA proportion, especially of n-3 FA, mainly due to the high content of C18:3 *cis*-9, *cis*-12, *cis-*15. The CAL contained 20.24 mg/g DM of total polyphenolic compounds and 19.6 mg/g DM of diterpenes. Among the various diterpenes present in CAL, acetoxy dihydroxy royleanone had the highest concentration (13.4 mg/g DM). The CAL also contained 4.78 mg/g DM of dihydroxyroyleanone. Among the polyphenols, luteolin-O-glucuronide was 4.34 mg/g DM, rosmarinic acid 3.35 mg/g DM, and caffeic acid 3.19 mg/g DM (Table 4).

**Table 4:** Identified contents of the phenolic acids, flavonoids, and diterpenes in CAL

Compounds	Content, mg/g DM
Siringing acid	0.26
Vanilic acid	0.06
Dihydroxy benzoic acid	0.19
Hydroxy benzoic acid	1.03
Caffeic acid	3.20
Dihydro ferulic acid-O-glucuronide	0.25
Luteolin-O-(hexosyl)	0.42
Luteolin-O-glucuronide	4.34
Ferulic acid	0.25
Rosmarinic acid derivative	0.34
Apigenin-O-glucuronide	2.89
Rosmarinic acid	3.36
Luteolin-O-(maloylglycosyl)	1.73
Apigenin derivative	0.88
Carnosci acid glucoside	0.08
Luteolin	0.18
Luteolin-O-(rhamnosyl-hexosyl)	0.15
Apigenin	0.12
3′.4′-Dimethoxy quercetin	0.14
Salvianolic acid C	0.27
Diterpene derivative	0.37
Salvianolic acid C derivative	0.12
5.7-Dihydroxy-4′.6-dimethoxy flavone	0.075
Dihydroxy kaurenoic acid	0.045
Trihydroxy-ent-kauranoic acid	0.02
Rosmanol	0.085
Dihydroxy kaurenoic acid	0.17
Longikaurin A	0.28
Dihydroxy royleanone	4.78
Epirosmanol	0.11
Dihydroxy-16-kauren-19-oic acid	0.10
Diterpene	0.22
Acetyl dihydroxy royleanone	13.41
Total phenolic acids	9.30
Total flavonoids	10.94
Total polyphenolic content	20.24
Total diterpenes	19.59

### In vitro experiment (Exp. 1)

Increased supplementation with CAL did not alter the basic ruminal fermentation parameters, such as pH and concentrations of ammonia or total VFA (Table 5). However, the molar proportions of acetate, propionate, butyrate, isovalerate, and valerate were dose-dependent. The proportions of acetate and isovalerate were lower in the 15% and 20% CAL treatments (*P* < 0.05), but the proportions of butyrate and valerate were higher in the CAL diets than in CON (*P* ≤ 0.02). A linearly lower (*P* < 0.01) A/P ratio was observed with increasing CAL. Digestibility of DM, OM, and NDF was unaffected by CAL, but a higher crude protein digestibility was noted for the 10% and 20% CAL supplementation with a linear response (*P* = 0.03). Total gas and CH_4_ production (mL or mL/g DM) decreased linearly (*P* < 0.02) with increasing levels of CAL in diets. Protozoa counts were unaffected by the CAL diet (Table 6). The 10% CAL addition increased the populations of *Streptococcus bovis, Prevotella* spp., *Butyrivibrio proteoclasticus,* and *Butyrivibrio fibrisolvens* (*P* ≤ 0.02). The CAL treatments linearly decreased the total Archaea and Methanobacteriales populations (*P* < 0.01).

**Table 5:** The effect of CAL on in vitro ruminal fermentation and methane production (Exp. 1)

Parameters	CON	CAL, % DM			SEM	*P*-value	
		10	15	20		Diet	L
Rumen fermentation							
Redox potential, mV	− 335	− 336	− 329	− 333	2.11	0.35	0.34
pH	6.89	6.89	6.91	6.91	0.001	0.12	0.08
NH_3_, mmol/L	9.19	9.19	9.11	9.18	0.25	0.99	0.96
Total VFA, mmol/L	44.7	44.4	44.6	47.1	1.15	0.62	0.36
VFA, molar percent							
Acetate (A)	61.3^*a*^	59.7^*ab*^	58.2^*b*^	56.9^*c*^	0.74	0.01	< 0.01
Propionate (P)	22.9^*bc*^	22.7^*c*^	23.7^*ab*^	24.7^*a*^	0.37	< 0.01	< 0.01
Isobutyrate	3.36	3.56	3.57	3.62	0.17	0.78	0.35
Butyrate	8.19^*b*^	9.10^*a*^	9.36^*a*^	9.36^*a*^	0.14	0.02	< 0.01
Isovalerate	1.01^*ab*^	1.12^*a*^	0.91^*b*^	0.93^*b*^	0.03	0.04	0.13
Valerate	3.01^*c*^	3.76^*b*^	4.15^*ab*^	4.22^*a*^	0.14	< 0.01	< 0.01
A/P ratio	2.75^*a*^	2.69^*a*^	2.51^*b*^	2.35^*b*^	0.07	< 0.01	< 0.01
Digestibility, g/kg DM							
Dry matter	505	526	499	518	6.40	0.38	0.78
Organic matter	516	530	504	524	6.36	0.47	0.99
Crude protein	430^*b*^	489^*a*^	453^*ab*^	489^*a*^	7.82	0.01	0.03
Neutral detergent fiber	491	506	483	488	7.11	0.66	0.59
Total gas and methane emission							
Gas, mL	2902^*a*^	2984^*a*^	2920^*a*^	2534^*b*^	51.0	< 0.01	0.01
Gas, mL/g DM	264^*a*^	271^*a*^	265^*a*^	230^*b*^	4.63	< 0.01	0.01
CH_4,_ mL	92.0^*a*^	79.0^*ab*^	71.1^*b*^	46.6^*c*^	3.84	< 0.01	< 0.01
CH_4_, mL/g DM	8.64^*a*^	7.41^*ab*^	6.45^*b*^	4.22^*c*^	0.34	< 0.01	< 0.01
CH_4_, mL/L gas	33.6^*a*^	28.3^*ab*^	24.2^*b*^	18.2^*c*^	1.24	< 0.01	< 0.01
CH_4_, mL/g DMD	17.1^*a*^	14.0^*b*^	14.9^*ab*^	8.61^*c*^	0.70	< 0.01	< 0.01
CH_4_, mL/g OMD	16.8^*a*^	13.0^*ab*^	12.4^*b*^	8.90^*b*^	0.69	< 0.01	< 0.01
CH_4_, mL/g NDF	15.6	14.4	13.9	8.46	0.80	0.08	0.02

*CON* control diet, *CAL Coleus amboinicus* Lour. diet, *SEM* standard error of means, *L* linear response, *DM* dry matter, *NH*_*3*_ ammonia, *VFA* volatile fatty acid, *CH*_*4*_ methane, *DMD* dry matter digestibility, *OMD* organic matter digestiblity

Different superscripts (a, b, c) within the same row indicate significant differences (*P* < 0.05)

**Table 6:** The effect of CAL on in vitro ruminal microorganisms (Exp. 1)

Variables	CON	CAL, % DM			SEM	*P*-value	
		10	15	20		Diet	L
Total protozoa, 10^3^/mL	4.89	4.68	4.68	4.91	0.003	0.40	0.91
Holotricha, 10^3^/mL	0.05	0.04	0.04	0.05	0.06	0.72	0.55
Entodinomorpha, 10^3^/mL	4.85	4.64	4.64	4.87	0.07	0.39	0.91
Total bacteria, 10^8^/mL	4.74	4.31	4.51	4.48	0.06	0.08	0.27
Streptococcus bovis*	1.00^*ab*^	5.26^*a*^	0.08^*b*^	0.16^*b*^	0.70	0.01	0.76
Ruminococcus flavefaciens*	1.00	0.43	0.88	0.43	0.19	0.63	0.38
Ruminococcus albus*	1.00	0.10	3.16	18.5	3.08	0.13	0.09
Megasphaera elsdenii*	1.00	4.07	4.63	3.69	0.86	0.47	0.20
*Prevotella* spp.*	1.00^*b*^	8.54^*a*^	1.93^*ab*^	0.46^*b*^	1.06	0.02	0.84
*Lactobacillus* spp.*	1.00	0.05	0.33	0.11	0.05	0.12	0.22
Fibrobacter succinogenes*	1.00	0.57	0.28	0.02	0.10	0.27	0.40
Butyrivibrio proteoclasticus*	1.00^*b*^	5.42^*a*^	2.38^*ab*^	1.53^*b*^	0.54	< 0.01	0.47
Butyrivibrio fibrisolvens*	1.00^*b*^	15.0^*a*^	4.83^*ab*^	3.61^*ab*^	1.60	0.01	0.53
Anaerovibrio lipolytica*	1.00	0.23	0.81	0.34	0.13	0.12	0.15
Total methanogens, 10^7^/mL	4.39^*a*^	3.87^*b*^	3.91^*b*^	3.32^*c*^	0.10	< 0.01	< 0.01
Methanobacteriales, 10^6^/mL	3.05^*a*^	2.85^*b*^	2.81^*bc*^	2.67^*c*^	0.04	< 0.01	< 0.01
Methanomicrobiales, 10^6^/mL	3.06^*a*^	2.89^*b*^	2.97^*b*^	2.67^*ab*^	0.04	0.01	0.13

*CON* control diet, *CAL Coleus amboinicus* Lour. diet, *SEM* standard error of means, *L* linear response

*Relative transcript abundance (∆∆ CT)

Different superscripts (a, b, c) within the same row indicate significant differences (*P* < 0.05)

The FA proportions in the ruminal fluid were altered by CAL supplementation (Table 7). The C18:0, C18:1 *trans-*10, C18:1 *trans-*11, total C18:1 *trans*, C20:1 *trans*, decreased linearly with increasing levels of CAL in the diets (*P* < 0.05). The C18:1*cis-*9, C18:1*cis-*11, C24:1, C18:2 *cis-*12 *trans-*10 (CLA), C18:2 *cis-*9 *cis-*12 (linoleic acids; LA), PUFA, and n-6 FA proportions in CAL treatments increased linearly (*P* < 0.05). The CAL treatments had a lower total SFA and higher total UFA proportion in ruminal fluid, and both were altered in a linear manner (*P* < 0.01). Also, the total BH intermediates, LA-BH and LNA-BH were decreased by CAL with a linear response (*P* < 0.01). The C18:3 *cis-*9 *cis-*12 *cis-*15, n-3 FA, total CLA, PUFA/SFA and LNA/LA proportions were higher at 20% CAL supplementation (*P* ≤ 0.05) than the CON.

**Table 7:** The effect of CAL on in vitro ruminal FA composition (Exp. 1)

Fatty acid, % of total FA	CON	CAL, % DM			SEM	*P*-value	
		10	15	20		Diet	L
Saturated FA							
C10:0	0.26	0.31	0.30	0.28	0.02	0.84	0.77
C12:0	1.33	1.30	1.62	1.42	0.07	0.28	0.29
C14:0	1.53	1.51	1.51	1.47	0.04	0.94	0.56
C15:0	1.14	1.13	1.10	1.09	0.03	0.91	0.49
C16:0	21.2	21.3	21.7	20.9	0.20	0.48	0.89
C17:0	0.76	0.72	0.72	0.72	0.02	0.78	0.39
C18:0	41.0^*a*^	36.7^*b*^	36.5^*b*^	33.8^*b*^	0.77	< 0.01	< 0.01
C20:0	0.07	0.09	0.08	0.06	0.01	0.63	0.54
C21:0	0.20	0.21	0.18	0.19	0.02	0.95	0.77
C22:0	0.13	0.16	0.16	0.18	0.01	0.46	0.13
C23:0	0.17	0.16	0.14	0.15	0.01	0.82	0.43
C24:0	0.52	0.46	0.45	0.44	0.02	0.17	0.04
Monounsaturated FA							
C14:1	0.50	0.48	0.37	0.38	0.03	0.12	0.04
C15:1	0.52	0.62	0.55	0.60	0.03	0.69	0.57
C16:1	0.57	0.77	0.61	0.89	0.06	0.16	0.11
C17:1	0.21	0.23	0.25	0.24	0.02	0.90	0.47
C18:1 *trans-10*	1.01^*a*^	0.80^*ab*^	0.65^*b*^	0.57^*b*^	0.06	0.05	< 0.01
C18:1 *trans-11* (VA)	2.40^*a*^	1.84^*b*^	1.86^*b*^	1.67^*b*^	0.08	< 0.01	< 0.01
C18:1 *cis-9*	10.9^*b*^	12.9^*a*^	13.8^*a*^	13.7^*a*^	0.37	0.02	< 0.01
C18:1 *cis-11*	1.14^*b*^	1.26^*ab*^	1.33^*a*^	1.31^*a*^	0.03	0.03	< 0.01
C18:1 *cis-12*	0.14	0.14	0.11	0.12	0.01	0.68	0.37
C18:1 *cis-13*	0.20	0.14	0.09	0.14	0.02	0.11	0.09
C18:1 *cis-14*	0.33	0.36	0.37	0.37	0.02	0.85	0.42
C20:1 *trans*	0.95^*a*^	0.89^*ab*^	0.87^*ab*^	0.81^*b*^	0.02	0.05	< 0.01
C24:1	0.15^*b*^	0.16^*b*^	0.27^*a*^	0.28^*a*^	0.02	< 0.01	0.03
Polyunsaturated FA							
C18:2 *cis-12 trans-10*	0.23^*b*^	0.30^*a*^	0.23^*b*^	0.32^*a*^	0.01	< 0.01	0.04
C18:2 *cis-9 trans-11* (RA)	0.09	0.12	0.13	0.09	0.01	0.45	0.99
C18:2 *cis-9 cis-12* (LA)	10.5^*b*^	12.6^*a*^	12.3^*a*^	13.0^*a*^	0.35	0.04	0.02
C18:3 *cis-6 cis-9 cis-12*	0.12	0.09	0.10	0.11	0.01	0.43	0.84
C18:3 *cis-9cis-12cis-15* (LNA)	1.25^*b*^	1.79^*ab*^	1.59^*ab*^	4.10^*a*^	0.35	0.01	< 0.01
C20:2	0.32	0.31	0.32	0.28	0.03	0.96	0.72
C22:2	0.12	0.09	0.10	0.11	0.01	0.65	0.75
∑ SFA	68.3^*a*^	64.1^*b*^	64.4^*b*^	60.7^*c*^	0.79	< 0.01	< 0.01
∑ UFA	31.7^*a*^	35.9^*b*^	35.6^*b*^	39.2^*c*^	0.79	< 0.01	< 0.01
∑ MUFA	19.0	20.6	20.8	21.2	0.38	0.15	0.04
∑ PUFA	12.6^*c*^	15.3^*b*^	14.8^*bc*^	17.9^*a*^	0.55	< 0.01	< 0.01
∑ n-6	11.1^*b*^	13.1^*a*^	12.8^*ab*^	13.5^*a*^	0.35	0.04	0.02
∑ n-3	1.25^*b*^	1.79^*b*^	1.59^*b*^	4.10^*a*^	0.35	0.01	< 0.01
n-6/n-3	9.11	8.75	8.98	6.94	0.44	0.26	0.11
PUFA/SFA	0.19^*b*^	0.25^*b*^	0.23^*b*^	0.31^*a*^	0.01	< 0.01	< 0.01
LNA/LA	0.12^*b*^	0.14^*b*^	0.13^*b*^	0.31^*a*^	0.03	0.01	< 0.01
∑ C18:1	16.0	17.5	17.9	17.9	0.38	0.21	0.06
∑ C18:1 *trans-*	3.33^*a*^	2.50^*b*^	2.59^*b*^	2.28^*b*^	0.09	< 0.01	< 0.01
∑ C18:1 *cis-*	12.8^*b*^	15.0^*a*^	15.3^*a*^	15.7^*a*^	0.40	0.03	< 0.01
∑ CLA	0.32^*b*^	0.42^*a*^	0.36^*ab*^	0.41^*a*^	0.02	0.05	0.11
∑ MCFA	27.0	27.4	27.8	27.1	0.27	0.69	0.83
∑ LCFA	73.0	72.6	72.2	72.8	0.27	0.72	0.69
BH Int	4.31^*a*^	3.57^*b*^	3.52^*b*^	3.31^*b*^	0.10	< 0.01	< 0.01
LA BH, %	64.6^*a*^	57.2^*b*^	56.1^*b*^	53.6^*b*^	1.26	< 0.01	< 0.01
LNA BH, %	94.2^*a*^	92.0^*a*^	91.5^*a*^	78.1^*b*^	1.88	< 0.01	< 0.01
RA/VA	0.05	0.08	0.07	0.07	0.01	0.24	0.44

*CON* control diet, *CAL Coleus amboinicus* Lour. diet, *SEM* standard error of means, *L* linear response, *VA* vaccenic acid, *RA* rumenic acid, *SFA* saturated fatty acids, *MUFA* monounsaturated fatty acids, *PUFA* polyunsaturated fatty acids, *LA* linoleic acid, *MCFA* medium-chain fatty acids, *LCFA* long-chain fatty acids, *BH int* biohydrogenation intermediates, *CLA* conjugated linoleic acids, *LA BH,* biohydrogenation of linoleic acid, *LNA BH* biohydrogenation of linolenic acid

Different superscripts (a, b, c) within the same row indicate significant differences at *P* < 0.05 and tended to significant at *P* < 0.10

### In vivo experiment

In Exp. 2, ruminal pH and ammonia concentration in cannulated lambs fed the CAL diet was higher than in the CON group (*P* < 0.01), and these variables were post-feeding time dependent (*P* < 0.01; Table 8). The total VFA concentration was similar in both diets and was time-dependent (*P* < 0.01). Butyrate, isovalerate, and valerate proportions decreased when CAL treatment was used (*P* < 0.01). Time-dependent variation was observed in almost all the individual VFA proportions. The ratio of acetate to propionate (A/P) decreased in both diet- and time-dependent manners (*P* < 0.01).

**Table 8:** The effect of CAL on ruminal fermentation in cannulated lambs (Exp. 2)

Parameters	0 h			3 h			6 h			Group		SEM	*P*-value		
	CON	CAL	SEM	CON	CAL	SEM	CON	CAL	SEM	CON	CAL		D	H	D × H
pH	6.87	7.05	0.09	5.95	6.39	0.06	6.34	6.58	0.04	6.39	6.67	0.05	< 0.01	< 0.01	0.20
NH_3,_ mmol/L	8.75	10.4	0.33	9.34	11.2	0.45	5.85	8.93	0.57	7.98	10.2	0.30	< 0.01	< 0.01	0.27
Total VFA, mmol/L	66.8	67.9	2.77	103.6	101.3	3.83	83.4	86.9	1.50	84.6	85.4	2.36	0.79	< 0.01	0.73
VFA, molar percent															
Acetate (A)	71.6	69.8	0.39	66.2	66.4	0.41	67.4	67.7	0.44	68.4	67.9	0.32	0.29	< 0.01	0.08
Propionate (P)	15.6 ^d^	17.5^c^	0.34	16.3 ^d^	20.1^a^	0.49	15.7^d^	18.9^b^	0.39	15.9	18.8	0.25	< 0.01	< 0.01	0.04
Isobutyrate	0.42	0.61	0.07	0.52	0.93	0.10	0.46	0.76	0.07	0.47	0.77	0.05	< 0.01	0.15	0.61
Butyrate	10.3^b^	10.5^b^	0.11	14.9^a^	11.1^b^	0.50	14.4^a^	11.2^b^	0.51	13.2	11.0	0.28	< 0.01	< 0.01	< 0.01
Isovalerate	1.00	0.75	0.05	0.53	0.41	0.02	0.62	0.44	0.03	0.72	0.53	0.03	< 0.01	< 0.01	0.31
Valerate	1.11^c^	0.92^d^	0.04	1.59^a^	1.07^cd^	0.07	1.36^b^	1.03^cd^	0.07	1.36	1.01	0.04	< 0.01	< 0.01	0.01
A/P ratio	4.61	4.04	0.11	4.07	3.33	0.10	4.29	3.61	0.09	4.33	3.66	0.06	< 0.01	< 0.01	0.71

*CON* control diet, *CAL Coleus amboinicus* Lour. diet, *SEM* standard error of means. *D* diet, *H* hour, *VFA* volatile fatty acids

Means in the same row indicate significant differences at *P* < 0.05 and tended to significant at *P* < 0.10

Holotricha had a lower population in the CAL group than in the CON group (*P* = 0.02; Table 9). Entodiniomorpha and total protozoa tended to increase (*P* = 0.07; *P* = 0.06, respectively) due to CAL supplementation. The CAL diet tended to increase total bacteria abundance (*P* = 0.09, Table 9). Populations of all methanogens, Methanobacteriales and Methanomicrobiales were decreased by the CAL diet (*P* ≤ 0.04).

**Table 9:** The effect of CAL on ruminal microbial populations in cannulated lambs (Exp. 2)

Parameters	Control	CAL	SEM	*P*-value
Total protozoa, 10^5^/mL	4.43	5.76	0.42	0.06
Holotricha, 10^5^/mL	0.08	0.04	0.01	0.02
Entodiniomorpha, 10^5^/mL	4.35	5.72	0.42	0.07
Total bacteria, 10^9^/mL	4.12	4.37	0.07	0.09
Total methanogens, 10^8^/mL	5.34	4.17	0.18	< 0.01
Methanobacteriales, 10^7^/mL	4.20	3.03	0.16	< 0.01
Methanomicrobiales, 10^7^/mL	3.35	2.97	0.09	0.04

*CON* control diet, *CAL Coleus amboinicus* Lour. diet, *SEM* standard error of means

Means in the same row indicate significant differences at *P* < 0.05 and tended to significant at *P* < 0.10

In Exp. 3, the CAL diet did not affect performance of growing lambs or feed digestibility (Table 10), but significantly lowered the CH_4_ production expressed as a L/d, L/kg DMI, L/kg OM (*P* < 0.01), and CH_4_/BW (*P* = 0.02). The pH value was higher (*P* = 0.05) and ammonia concentration tended to increase (*P* = 0.09), when the CAL diet was used. Total VFA concentration did not change, but the propionate proportion was higher (*P* = 0.01) in the CAL group than in CON group. The concentrations of isovalerate and valerate as well as the A/P ratio were lower (*P* ≤ 0.04) in CAL group than the CON group.

**Table 10:** Impact of CAL on performance, methane emission, and ruminal fermentation of lambs (Exp. 3)

Parameters	CON	CAL	SEM	*P*-value
Body weigth, kg				
Inital BW	19.3	19.7	0.66	0.70
Final BW	25.1	26.8	0.53	0.13
ADG, g/d	167	196	9.45	0.14
Total tract digestibility, g/kg				
Dry matter	630	649	8.27	0.26
Organic matter	607	604	6.80	0.82
Crude protein	604	601	7.71	0.85
Neutral detergent fiber	577	578	13.3	0.97
Methane emission				
CH_4_, L/d	20.0	15.9	0.11	< 0.01
CO_2_, L/d	307	274	2.24	< 0.01
CH_4_/CO_2_, mL/L	63.6	54.4	0.26	< 0.01
CH_4_, L/kg DM intake	29.9	21.1	1.44	< 0.01
CH_4_, L/kg OM intake	32.4	23.3	1.49	< 0.01
CH_4_, L/kg BW	1.02	0.82	0.05	0.02
Rumen fermentation				
pH	6.22	6.35	0.04	0.05
NH_3_, mmol/L	9.92	15.5	0.64	0.09
Total VFA, mmol/L	112	118	3.70	0.48
VFA, molar percent				
Acetate (A)	69.9	68.1	0.51	0.07
Propionate (P)	17.7	19.8	0.46	0.01
Isobutyrate	0.95	0.79	0.05	0.08
Butyrate	9.92	10.2	0.30	0.70
Isovalerate	0.47	0.23	0.05	< 0.01
Valerate	1.06	0.86	0.05	0.04
A/P ratio	3.96	3.46	0.11	0.01

*CON* control diet, *CAL Coleus amboinicus* Lour. diet, *SEM* standard error of means, *DM* dry matter, *OM* organic matter, *BW* body weight, *ADG* average daily gain, *VFA* volatile fatty acids

Means in the same row indicate significant differences at *P* < 0.05 and tended to significant at *P* < 0.10

The protozoa population was increased (*P* < 0.01) by the CAL diet (Table 11) as a result of the increased population of Entodiniomorpha; but Holotricha population decreased (*P* < 0.01). *M. elsdenii* and *B. proteoclasticus* were higher (*P* ≤ 0.02) in the CAL group than the CON group. Numbers of *R. albus*, *Prevotella* spp., and *B. fibrisolvens* tended to increase (*P* = 0.06; *P* = 0.10; *P* = 0.09, respectively) with the CAL diet. The total methanogens and Methanobacteriales decreased (*P* < 0.01 and *P* = 0.05, respectively) due to CAL supplementation.

**Table 11:** The effect of CAL on ruminal microbial populations in lambs (Exp. 3)

Parameters	CON	CAL	SEM	*P*-value
Total protozoa, 10^5^/mL	6.47	8.43	0.45	< 0.01
*Holotricha,* 10^5^/mL	0.05	0.02	0.01	< 0.01
*Entodinomorpha,* 10^5^/mL	6.42	8.41	0.46	< 0.01
Total bacteria, 10^9^/mL	4.89	4.77	0.09	0.55
Streptococcus bovis*	1.01	0.83	0.26	0.75
Ruminococcus flavefaciens*	1.00	0.94	0.24	0.91
Ruminococcus albus*	1.00	4.71	0.92	0.06
Megasphaera elsdenii*	1.00	7.68	1.44	0.03
*Prevotella* spp.*	1.00	3.85	0.86	0.10
*Lactobacillus* spp.*	1.00	0.10	0.47	0.38
Fibrobacter succinogenes*	1.00	5.60	1.89	0.24
Butyrivibrio proteoclasticus*	1.00	15.9	3.03	0.02
Butyrivibrio fibrisolvens*	1.00	4.55	1.06	0.09
Total methanogens, 10^8^/mL	5.08	3.61	0.28	< 0.01
Methanobacteriales, 10^7^/mL	3.23	2.73	0.12	0.05
Methanomicrobiales, 10^7^/mL	3.08	3.00	0.13	0.75

*CON* control diet, *CAL Coleus amboinicus* Lour. diet, *SEM* standard error of means

*Relative transcript abundance (∆∆ CT)

Means in the same row indicate significant differences at *P* < 0.05 and tended to significant at *P* < 0.10

In Exp. 3, the proportions of C16:0, C23:0, C24:1, LNA, PUFA, the sum of n-6, and the sum of n-3 FA in ruminal fluid were increased by the CAL diet (*P* ≤ 0.05, Table 12). The CAL diet significantly decreased the proportions of C18:0 andC18:1 *trans-10* (*P* < 0.05). Total SFA and PUFA/SFA tended to decrease while the total UFA tended to increase (*P* < 0.10). The ruminal biohydrogenation percentage of LA and LNA decreased in the CAL diet (*P* < 0.05). Stearic acid (C18:0), sum of SFA, thrombogenicity index (TI), and the atherogenicity index (AI) were decreased (*P* ≤ 0.05) in LT muscle by CAL diet. The proportions of C18:3 *cis-*9 *cis-*12 *cis-*15, sum of UFA, PUFA/SFA, total CLA, D∆9, D∆9 18:1/18:0, D∆9 RA/VA increased (*P* ≤ 0.05) in the muscle for the CAL diet. The proportions of total PUFA and total n-3 FA in LT muscle tended to increase (*P* = 0.07; *P* = 0.07; *P* = 0.08, respectively) when the CAL diet was used.

**Table 12:** Fatty acid profile in rumen fluid and *longissimus thoracis* muscle of lambs fed CAL (Exp. 3)

Fatty acid, % of total FA	Rumen fluid				LT muscle			
	CON	CAL	SEM	*P* value	CON	CAL	SEM	*P*-value
Saturated FA								
C8:0	0.03	0.01	0.01	0.39	1.44	0.60	0.39	0.31
C10:0	0.08	0.49	0.14	0.16	1.85	1.49	0.41	0.68
C12:0	0.64	0.98	0.13	0.19	1.62	1.23	0.26	0.48
C13:0	1.87	1.94	0.17	0.86	1.09	0.82	0.26	0.63
C14:0	1.11	1.29	0.14	0.56	1.77	1.62	0.26	0.79
C15:0	1.46	1.44	0.13	0.92	1.13	1.11	0.22	0.96
C16:0	16.5	18.7	0.49	0.01	14.1	12.5	0.50	0.11
C17:0	0.73	0.62	0.07	0.48	0.87	1.07	0.13	0.48
C18:0	47.3	40.9	1.59	0.03	15.4	12.1	0.73	0.01
C20:0	0.06	0.06	0.02	0.86	0.21	0.20	0.04	0.88
C21:0	0.05	0.11	0.02	0.17	0.26	0.38	0.07	0.41
C22:0	0.11	0.08	0.02	0.37	0.52	0.65	0.08	0.44
C23:0	0.09	0.21	0.03	0.02	0.34	0.71	0.11	0.10
C24:0	0.56	0.55	0.03	0.87	0.18	0.50	0.09	0.09
Monounsaturated FA								
C14:1	0.24	0.28	0.06	0.76	0.69	0.96	0.17	0.45
C15:1	1.11	0.87	0.12	0.32	1.57	1.49	0.20	0.84
C16:1	0.31	0.32	0.05	0.96	1.03	0.99	0.14	0.89
C17:1	0.17	0.17	0.05	0.99	1.24	1.71	0.18	0.20
C18:1 *trans-10*	0.62	0.48	0.04	0.05	0.30	0.21	0.03	0.12
C18:1 *trans-11* (VA)	5.25	4.41	0.30	0.19	1.07	0.75	0.10	0.11
C18:1 *cis-9*	6.98	7.53	0.37	0.49	22.1	22.4	1.11	0.92
C18:1 *cis-11*	1.65	1.68	0.06	0.83	2.30	2.17	0.12	0.62
C18:1 *cis-12*	0.57	0.68	0.06	0.46	0.77	0.62	0.08	0.37
C18:1 *cis-13*	0.12	0.19	0.03	0.26	0.23	0.21	0.05	0.86
C18:1 *cis-14*	1.00	0.81	0.10	0.36	0.63	0.50	0.11	0.57
C20:1 *trans-*	0.84	0.90	0.03	0.36	0.52	0.64	0.11	0.64
C22:1 *n-9*	0.16	0.09	0.03	0.22	0.38	0.60	0.09	0.23
C24:1	0.14	0.71	0.11	< 0.01	1.44	1.92	0.14	0.10
Polyunsaturated FA								
C18:2 *cis-12 trans-10* (CLA-*t10*)	0.17	0.66	0.15	0.18	0.51	0.56	0.10	0.82
C18:2 *cis-9 trans-11* (RA)	0.49	0.37	0.05	0.21	0.44	0.69	0.11	0.27
C18:2 *cis-9 cis-12* (LA)	6.42	8.07	0.40	0.02	15.1	17.6	0.93	0.19
C18:3 *cis-6 cis-9 cis-12*	0.15	0.27	0.04	0.11	0.49	0.57	0.09	0.67
C18:3 *cis-9 cis-12 cis-15* (LNA)	1.47	2.28	0.16	< 0.01	1.05	1.73	0.19	0.05
C20:2	0.43	0.37	0.06	0.63	0.35	0.44	0.10	0.68
C20:3 n-6	0.63	0.65	0.07	0.89	4.04	5.11	0.38	0.17
C20:4 n-6	nd.	nd.	nd.	nd.	0.51	0.42	0.05	0.44
C20:5 n-3	nd.	nd.	nd.	nd.	0.74	0.64	0.14	0.73
C22:2	0.08	0.16	0.04	0.28	0.62	0.49	0.15	0.72
C22:5 n-3	nd.	nd.	nd.	nd.	0.64	0.90	0.09	0.17
C22:6 n-3	nd.	nd.	nd.	nd.	0.45	0.79	0.15	0.27
∑ SFA	70.5	67.3	0.96	0.10	40.8	34.9	1.24	0.01
∑ UFA	29.5	32.7	0.96	0.10	59.2	65.1	1.24	0.01
∑ MUFA	18.7	20.1	0.60	0.66	34.3	35.1	0.95	0.70
∑ PUFA	10.8	12.5	0.51	< 0.01	24.9	30.0	1.01	0.07
∑ n-6	8.90	9.72	0.42	0.02	21.6	25.2	1.23	0.17
∑ n-3	1.47	2.28	0.16	< 0.01	2.88	4.06	0.31	0.07
n-6/n-3	6.05	4.37	0.39	0.02	7.86	6.74	0.73	0.47
PUFA/SFA	0.15	0.19	0.01	0.07	0.62	0.86	0.05	0.01
LNA/LA	0.21	0.30	0.02	0.02	0.07	0.10	0.01	0.31
∑ C18:1	15.7	16.8	0.64	0.45	27.4	26.8	1.22	0.81
∑ C18:1 *trans-*	5.48	5.08	0.28	0.52	1.37	0.96	0.11	0.06
∑ C18:1 *cis-*	10.2	11.7	0.58	0.23	26.1	25.8	1.19	0.94
∑ CLA	0.64	0.99	0.19	0.36	0.95	1.61	0.18	0.04
∑ MCFA	23.3	26.3	0.94	0.12	26.3	22.8	1.25	0.17
∑ LCFA	76.7	73.7	0.94	0.12	73.7	77.2	1.25	0.17
BH intermediates	7.81	7.76	0.46	0.95	nd.	nd.	nd.	nd.
LA BH, %	74.2	67.6	1.58	0.03	nd.	nd.	nd.	nd.
LNA BH, %	94.4	91.5	0.61	< 0.01	nd.	nd.	nd.	nd.
RA/VA	0.08	0.11	0.02	0.50	nd.	nd.	nd.	nd.
Desaturation index (DI) ∆9	nd.	nd.	nd.	nd.	0.41	0.47	0.01	0.01
D∆9. C14:1/C14:0	nd.	nd.	nd.	nd.	0.29	0.37	0.06	0.50
D∆9. 16:1/16:0	nd.	nd.	nd.	nd.	0.07	0.07	0.01	0.85
D∆9. 18:1/18:0	nd.	nd.	nd.	nd.	0.59	0.65	0.01	0.01
D∆9. RA/VA	nd.	nd.	nd.	nd.	0.27	0.48	0.06	0.04
D∆9. MUFA/SFA	nd.	nd.	nd.	nd.	0.46	0.50	0.01	0.08
D∆5. n-6. 20:4n-6/20:3n-6	nd.	nd.	nd.	nd.	0.12	0.08	0.01	0.11
D∆5. D6. n-6. 20:4n-6/18:3n-6	nd.	nd.	nd.	nd.	0.53	0.43	0.06	0.43
D∆4. n-3. 22:6n-3/22:5n-3	nd.	nd.	nd.	nd.	0.33	0.41	0.06	0.53
Elongase index	nd.	nd.	nd.	nd.	0.71	0.72	0.01	0.92
Thrombogenic index	nd.	nd.	nd.	nd.	0.85	0.62	0.05	< 0.01
Atherogenicity index	nd.	nd.	nd.	nd.	0.63	0.48	0.03	0.03

*CON* control diet, *CAL Coleus amboinicus* Lour. diet, *SEM* standard error of means, *SFA* saturated fatty acids, *UFA* unsaturated fatty acids, *MUFA* monounsaturated fatty acids, *PUFA* polyunsaturated fatty acids, *BH* biohydrogenation, *LNA* linolenic acid, *RA* rumenic acid, *VA* vaccenic acid, *LA* linoleic acid, *MCFA* medium-chain fatty acids, *LCFA* long-chain fatty acids, *nd.* not determined

Means in the same row indicate significant differences at *P* < 0.05 and tended to significant at *P* < 0.10

**Fig. 1:**
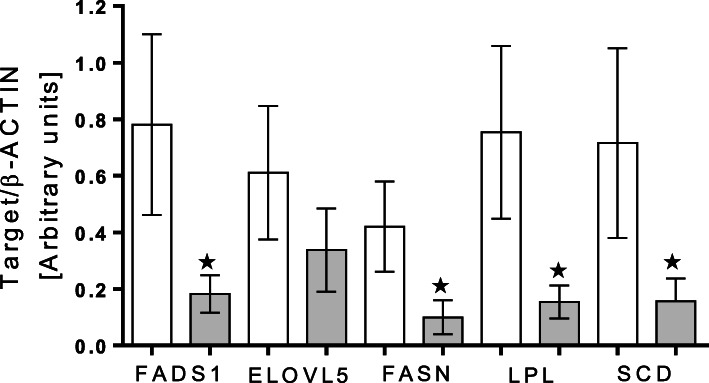
Comparison of gene expressions in *longissimus thoracis* muscle of lambs receiving CON and CAL diet. Legends: (CON; white colour); CAL diet (CAL; grey colour); Gene expressions are fatty acid desaturase 1 *(FADS1),* fatty acid elongase 5 *(ELOVL5),* fatty acid synthase *(FASN),* lipoprotein lipase *(LPL),* and stearoyl-CoA desaturase *(SCD)* in the *longissimus thoracis* muscle of growing lambs. The symbol * indicates significant difference between treatments (*P* *<* 0.05)

The CAL diet significantly decreased the mRNA expressions of *FADS1, FASN, LPL,* and *SCD* genes, but not the expression of *ELOVL5* (Fig. 1). The CAL diet also significantly affected some meat characteristics. Lightness color and water-holding capacity of meat were reduced, whereas meat juiciness was increased (*P* ≤ 0.01, Table 13) by CAL diet. Redness and flavor values tended to increase (*P* = 0.09; *P* = 0.06, respectively), whereas the ash content tended to decrease due to CAL supplementation (*P* = 0.09).

**Table 13:** The *longissimus thoracis* muscle quality and characteristics of lambs fed CAL (Exp. 3)

Trait	Unit	Treatments		SEM	*P*-value
		CON (*n* = 8)	CAL (*n* = 8)		
Muscle pH 24 h post mortem	pH units	5.58	5.69	0.05	0.39
L*, lightness	units	41.4	37.0	0.88	0.01
a*, redness	units	12.1	13.2	0.31	0.09
b*, yellowness	units	4.81	4.65	0.36	0.84
Water-holding capacity, %		38.6	29.5	1.69	< 0.01
Watercontent, %		72.9	73.3	0.24	0.40
Intramuscular fat content, %		2.58	2.36	0.07	0.13
Total protein content, %		23.3	23.0	0.25	0.58
Ash content, %		1.16	1.13	0.01	0.09
Sensory evaluations					
Aroma	1–5	4.28	4.29	0.05	0.85
Juiciness	1–5	4.19	4.57	0.08	0.01
Tenderness	1–5	4.15	4.36	0.07	0.13
Flavor	1–5	4.07	4.31	0.07	0.06
Subjective visual evaluations					
Color	1–8	2.38	2.56	0.10	0.38
Marbling	1–4	1.59	1.53	0.06	0.63

*CON* control diet, *CAL Coleus amboinicus* Lour. diet, *SEM* standard error of means

Means in the same row indicate significant differences at *P* < 0.05 and tended to significant at *P* < 0.10

## Discussion

### Nutrients and phytochemical composition of *Coleus amboinicus* Lour

Several studies confirmed that CAL is rich in nutrients, ash, and BACs [6, 7]. In comparison to previous study [7], the CAL examined in this experiment was characterized by higher concentrations of protein (214 vs. 196 g/kg DM), aNDF (405 vs. 363 g/kg DM), *α*-linolenic acid (45.1 vs. 35.83 g/100 g FA), and total PUFA (59.2 vs. 56.15 g/100 g FA). Regarding BACs, in this study similar concentrations of phenolic acids (9.30 mg/g DM) were detected as in Yanza et al. [6], whereas flavonoids level was four times higher (10.94 mg/g DM). Thus, total polyphenolic content equaled 20.24 mg/g DM. In case of diterpenes, their content equaled 19.79 mg/g DM and was nine times higher compared to Yanza et al. [6] study. The most abundant component of CAL was acetyl dihydroxy royleanone (13.4 mg/g DM), unlike in the study of Yanza et al. [6] where rosmarinic acid dominated. The differences between these results and those of Yanza et al. [6] may reflect characteristics of the plant materials such as harvesting time (2–3 months and 4–6 months, respectively) or management of plantation (e.g., watering and fertilizer application) [7]. The CAL used in this study had higher BAC content than that in Yanza et al. [6] and thus had a more pronounced action in the rumen.

### In vitro experiment (10 d RUSITEC fermentation, Exp. 1)

The gradual replacement of concentrate by CAL (from 10% to 20%) decreased methane production and diminished population of ruminal methanogens. Reduction in total methanogens (by 24%) and mitigation of CH_4_ production (by 51%; CH_4_/DM substrate) were observed for the 20% CAL content. Likewise, 20% of CAL in the diet effectively decreased CH_4_ production expressed as CH_4_/digestible DM, CH_4_/digestible OM and CH_4_/digestible NDF by 47%, 50%, and 45% respectively. We have noted the inhibition of methanogens via a direct toxic antibacterial effect of BAC, but without adverse effects on ruminal fermentation or DM degradability. Despite the toxic action of BAC on methanogens, no effect on protozoa and ruminal bacteria was observed. Only the lowest level of CAL (10%) increased the population of the bacteria *Prevotella* spp., *B. proteoclasticus*, and *B. fibrisolvens.* A distinct effect of the lowest CAL supplementation was also observed in our previous study on *Saponaria officinalis* [26, 27]. We hypothesized that the basic nutrient components of a plant can either interact with BAC or become physically less available for microbiota, resulting in a decreased antibacterial activity. Moreover, the antimethanogenic effect of CAL reduced methanogen population which in turn lowered the A:P ratio, probably by shifting the free-hydrogen pathways to propionate production.

*Coleus amboinicus* (Lour.) is also a rich source of UFA with the predominance of *α*-linolenic acid (LNA). The higher fat concentration in CAL than in grass silage (43 vs 21 g/kg DM, respectively) may increase the ruminal FA content, and consequently that of LNA. The effect of CAL in the diet on FA profile in the rumen differed from that on bacteria population. Dietary CAL modulated the ruminal FA composition by lowering SFA and elevating MUFA and PUFA proportions. The alterations in MUFA and PUFA profiles suggest the changes in BH process in the rumen. Based on the previous study [6], we had assumed that polyphenol compounds of CAL origin (flavonoid, phenolic acids, and diterpenes) can alter the action of the ruminal microbiota involved in BH. For instance, the CAL diet decreased C18:1 *trans* concentration due to the BH process of LA. High quantities of polyphenols and diterpenes reduced the final BH step of the C18:1 *cis-*9 and caused a linear decrease in the concentration of stearic acid (C18:0). A similar phenomenon was observed by Vasta et al. [3], who described a negative effect of different polyphenols on C18:0 accumulation in the rumen digesta. Hence, current study results were demonstrate the protective action of CAL on BH of MUFA and PUFA.

### In vivo experiments: Exp. 2 with cannulated lambs and Exp. 3 with growing lambs

Ruminal fermentation parameters of growing lambs receiving 20% of CAL corroborated the results of the current in vitro study. The CAL diet containing 4.08 g of total polyphenols per kg of DM and 3.96 g of total diterpenes per kg of DM, mitigated methane emission (L/d and L/DM intake) by 20% and 29%, respectively, and did not interfere with DM, OM, or NDF digestibility. Decreases in CH_4_ production are usually associated with adverse effects on fiber digestibility [28]. However, CH_4_ mitigation was directly linked to the reduction in total methanogen and Methanobacteriales (in both, cannulated and growing lambs) as well as in Methanomicrobiales populations (in the cannulated lambs) rather than to the reduction of carbohydrate digestibility [25].

Supplementing the diet with 20% of CAL had a positive effect on the total protozoa in the rumen of growing lambs. The effect was also more pronounced for a few particular bacterial species (namely *R. albus, M. elsdenii, B. proteoclasticus,* and *B. fibrisolvens*) than for the total bacteria. Increases in the number of several ruminal bacteria in heifers due to supplementation of BACs like flavonoids were also reported by De Nardi et al. [29]. On the other hand, diet formula affects qualitative and quantitative compositions of the rumen microbial population. Higher DM intake of grass silage in CAL diet (507 g/d/animal; 379 g/d/animal in CON) caused the rise of fibrolytic bacteria and protozoa populations. Such phenomenon elucidates coordinated activity of bacteria such as *F. succinogenes, R. albus,* and *B. fibrisolvens* and protozoa for proper fiber digestion [3, 30]. However, in the present study, neither digestibility nor total VFA concentration was affected, although alterations in individual VFAs, such as propionic acid, were noted. The high proportion of propionate was not linked with the abundance of *S. bovis*, the amylolytic bacteria responsible for production of this VFA. The possibility cannot be however excluded that other amylolytic bacteria, not investigated in this study, were not affected. Moreover, changes in nitrogen metabolizing bacteria population i.e., *B. proteoclasticus* and *B. fibrisolvens* were observed. It should be emphasized that CAL is rich in total protein (214 g/kg DM), and thus may be a perfect substrate for N metabolism in the rumen. The excessive protein of CAL origin may provide a high soluble fraction of total protein to the ration, which can directly stimulate ammonia production by the rumen bacteria, a phenomenon also observed in the present study.

In the in vivo experiments, higher values of ruminal pH were noticed. This result corroborated that of Balcells et al. [31] who reported higher pH after supplementing dairy cow diet with flavonoids. Microbial activity is affected by the properties of the substrate, and higher starch levels in the diet usually reduce the pH of the rumen fluid [32]. According to Balcells et al. [31], flavonoid supplementation may improve rumen fermentation and reduce the incidence of rumen acidosis. The present study confirmed this activity of CAL flavonoids (2.21 g/kg DM) by demonstrating increased pH values in the lamb rumen. It should be realized that the CAL contained three types of BAC with distinct activities: flavonoids, diterpenes and phenolic acids but the mechanism described above is typical for flavonoids. Such flavonoid activity partially explains the findings of the present study on the increased number of lactate-consuming microorganisms (e.g., *M. elsdenii*). Another mechanism that may stabilize the pH is the fluctuations of protozoa community. According to Hartinger et al. [33], protozoa incorporate starch granules that are not metabolized to organic acids quickly. Thus, protozoa prevent significant pH drops and support stable fermentation conditions in the rumen. In the present study, the *Entodiniomorpha* population significantly increased (Table 11), whereas that of Holotricha diminished consistently (Tables 9 and 11). Similar trends were described in short-term study [6], so we can assume that this mode of CAL action is stable for a longer period.

CAL diet positively altered ruminal PUFA proportion in the growing lambs, which can mainly be attributed to the increase in C18:3 *cis*-9 *cis*-12 *cis*-15 FAs in the rumen fluid and meat (Table 12). Although the majority of bacterial communities involved in BH increased in number, BH of C18 UFA into stearic acid (C18:0) was lower due to CAL intake. A similar result was reported by Vasta et al. [3], who, regardless of the type of polyphenol supplementation, observed a reduction in ruminal C18:0 and an increase in C18:1 *trans*-11 (VA) and C18:2 *cis*-9 *trans*-11 (CLA) isomers. The stability of the main BH isomers and the distinct responses of two bacteria species involved in BH process (*B. fibrisolvens* and *B. proteoclasticus*) observed in the present study can also demonstrate some other properties of polyphenols. The reduction of BH by CAL diet despite greater populations of BH bacteria may suggest that CAL may reduce the content of free FA, substrates to BH process due to decreased lipolysis [2]. Besides, a reduction in the numbers of several bacteria species involved in BH, such as *Neisseria weaverii*, *R. amylophilus*, and other unclassified bacteria related to the *Lachnospiraceae* and *Pasteurellaceae* families were observed [34]. In this study, the potential restriction on BH may be supported by the fact that the rumen and meat displayed similar alterations in the FA profile, which likely suggests that the rumen was the locus of the main changes.

The oxidative process can shorten the shelf life of fresh meat and negatively affect its consumption by the formation of off-flavors and discoloration [35]. The sensor attributes, likes juiciness and taste, are generally associated with the consumers’ preferences [36]. In this study, we observed improved meat characteristics such as juiciness or taste. The better meat sensory quality (Table 13) combined with improved indices of thrombogenicity (TI) and atherogenicity (AI) indexes (Table 12) may help to encourage consumers to select quality meat in the future. Tannin-containing diets sometimes reduce [37] or unchanged [38] tenderness and juiciness of meat depending upon the doses. In this study, juiciness and flavor of lamb meat increased, which would be advantageous from consumer perspectives. It has been suggested that phenolic compounds may stimulate calpain activity and thus degradation of myofibrillar proteins in carcass *postmortem*, leading to increased juiciness and tenderness of meat [39, 40]. Moreover, polyphenol inclusion in ruminants’ diet may cause different effects on the meat color. Priolo et al. [41] evaluated the effect of tannin in lambs and stated that the *longissimus* muscle was lighter (lower L*) in tannin-fed group. The lower lightness and greater redness of color in the present study were influenced by the CAL mineral content, which was not determined in this study. Damanik et al. [42] reported that the CAL contains high concentration of iron (Fe). Garg et al. [43] stated that the inclusion of tannin in the animal diets do not hamper the utilization of iron for the hemoglobin synthesis. Moreover, polyphenols with high antioxidant activities may reduce the oxidation of myoglobin leading to increase the redness of meat [35]. Increased redness of meat has also been reported due to feeding of polyphenol-rich plants [40, 44].

Transcript analysis performed in the present study included a panel of five genes of known functions in FA metabolism in ruminant muscles [45]. Three of the genes (*FASN, SCD,* and *ELOVL5*) control the de novo synthesis and elongation of FA, whereas the *LPL* and *FADS1* genes are involved in FA transport*.* Meat from lambs fed the CAL diet was characterized by a significant reduction in the mRNA content of four genes (*FASN, SCD*, *LPL,* and *FADS1*), whereas no changes were observed for the *ELOVL5* transcript. However, considering the complexity of the entire gene expression processes and the great variations in transcript lifespans, any conclusions must be drawn with caution. Alterations in the transcript expression of genes regulating lipid metabolism was not reflected in the profile of FA controlled by those genes. For example, the reduced mRNA level of the *SCD* gene was not accompanied by a lower level of C18:1 *cis*-9. According to Garnsworthy et al. [46], the level of C18:1 *cis*-9 in the fat of ruminant products is highly dependent upon the *SCD* gene controlling de novo FA synthesis. Besides, the lack of difference in C18:1 *cis-*9 concentration in response to CAL diet containing 4.08 g of total polyphenols per kg of DM and 3.96 g of total diterpenes per kg of DM may suggest that the synthesis of endogenous FA was unaffected. On the other hand, the reduced transcript levels of the *LPL* gene may be associated with the decreased biosynthesis of MUFA. The lack of alteration in the mRNA content of the *ELOVL5* gene regulating FA elongation and the increased n-3 concentration (mainly LNA) in meat may reflect n-3 metabolism in the rumen, rather than that in muscles. The reduced transcript content of another two genes -*FADS1* and *FASN*- may be linked to the inhibition of the initial stages of the BH process. Pewan et al. [47] suggested that the FASN protein complex controls de novo biosynthesis of long-chain FA and affects FA deposition in meat, adipose tissue, and milk. The published evidence on the correlations between n-3 PUFA profile, the activity of lipogenic genes (such as *FASN*), and meat quality, however, is very limited [47]. Nevertheless, the n-3 PUFA profile of the meat of lambs fed CAL was improved, which suggests that changes in the FA profile had already occurred in the rumen, leading to more PUFA being available to the tissue. Higher n-3 PUFA levels in meat are beneficial to human health and support cardiovascular, retinal, and brain functions [48, 49]. Positive changes in the FA profile of meat from the experimental lambs increased its quality but higher content of PUFAs could decrease the meat shelf life due to a rapid oxidization of FAs ensuing from two or more double bonds in their structure [50]. However, an important aspect is the improvement of *postmortem* characteristics and nutritional quality of meat from lambs fed CAL supplemented diet.

## Conclusions

In conclusions, polyphenols of CAL origin reduce CH_4_ production, which is associated with diminished Archaea communities. Consistent effects of CAL polyphenols on the final products of ruminal fermentation and lowering the A/P ratio were noted in both in vitro and in vivo experiments. Moreover, the CAL diet increased lambs’ nutrient intakes with no alteration in ruminal digestibility. Such CAL mode of action also affected ruminal bacteria involved in fermentation and BH what caused an increase in LNA concentration and ultimately increase deposition of n-3 PUFA. Consequently, 20% of CAL improved the meat characteristics and nutritional quality without negative effects on rumen fermentation and growth performance. Considering the diverse properties of *Coleus amboinicus* Lour., it can be an alternative feed to reduce greenhouse gas emissions and to improve the quality of ruminant products. This study shows that the use of dietary CAL could be useful for sustainable ruminant production in tropical areas as well as other parts of world for improving nutrition, reducing environmental issues and improving ruminant-derived food products.

## Data Availability

The experimental datasets of the present study can be obtained from the corresponding author on reasonable request.

## Abbreviations

ADG: Average daily gain
AI: Atherogenicity
aNDF: Ash free NDF
A/P ratio: Acetate/Propionate ratio
BAC: Biologically active compounds
BH: Biohydrogenation
BW: Body weight
CAL: *Coleus amboinicus* Lour
CH_4_: Methane emission
CO_2_: Carbon dioxide emission
CLA: Conjugated linoleic acid
CP: Crude protein
D∆: Desaturation ∆ at –*n*
DAPI: 4,6-diamidino-2-phenylindole
DI: Desaturation index
DM: Dry matter
DMD: Dry matter digestibility
DMI: Dry matter intake
DNA: Deoxyribonucleic acids
EE: Ether extract
EI: Elongase index
ELOVL5: Fatty acid elongase 5
FA: Fatty acids
FADS1: Fatty acid desaturase 1
FASN: Fatty acid synthase
FISH: Fluorescence in situ hybridization
IVDMD: in vitro dry matter digestibility
LA: *α-*Linoleic acids
LCFA: Long chain fatty acids
LT: *Longissimus thoracis*
LNA: *α-*Linolenic acids
LPL: Lipoprotein lipase
MCFA: Medium chain fatty acids
mRNA: Messenger-RNA
MUFA: Monounsaturated fatty acids
NDFD: Neutral detergent fibre digestibility
NH_3_: Ammonia
OM: Organic matter
PBS: Phosphate-buffered saline
PUFA: Polyunsaturated fatty acids
qPCR: Quantitative PCR
RA: Rumenic acid
RTA: Relative transcript abundance (∆∆ CT)
RUSITEC: Rumen simulation technique
SCD: Stearoyl-CoAdesaturase
SFA: Saturated fatty acids
SPE: Solid phase extraction
TI: Thrombogenicity
UFA: Unsaturated fatty acids
VA: Vaccenic acid
VFA: Volatile fatty acids
